# Metal ion-dependent, reversible, protein filament formation by designed beta-roll polypeptides

**DOI:** 10.1186/1472-6807-7-63

**Published:** 2007-10-01

**Authors:** Andrew J Scotter, Meng Guo, Melanie M Tomczak, Margaret E Daley, Robert L Campbell, Richard J Oko, David A Bateman, Avijit Chakrabartty, Brian D Sykes, Peter L Davies

**Affiliations:** 1 Protein Engineering Network Centres of Excellence, 750 Heritage Medical Research Centre, Edmonton, AB, T6G 2S2, Canada; 2 Department of Biochemistry, Queen's University, Kingston, ON, K7L 3N6, Canada; 3 Department of Medical Biophysics, University of Toronto, ON, M5G 2M9, Canada; 4 Ontario Cancer Institute, University of Toronto, ON, M5G 2M9, Canada; 5 Department of Biochemistry, University of Alberta, Edmonton, AB, 6G 2H7, Canada; 6 Department of Anatomy and Cell Biology, Queen's University, Kingston, Ontario, K7L 3N6, Canada

## Abstract

**Background:**

A right-handed, calcium-dependent β-roll structure found in secreted proteases and repeat-in-toxin proteins was used as a template for the design of minimal, soluble, monomeric polypeptides that would fold in the presence of Ca^2+^. Two polypeptides were synthesised to contain two and four metal-binding sites, respectively, and exploit stacked tryptophan pairs to stabilise the fold and report on the conformational state of the polypeptide.

**Results:**

Initial analysis of the two polypeptides in the presence of calcium suggested the polypeptides were disordered. The addition of lanthanum to these peptides caused aggregation. Upon further study by right angle light scattering and electron microscopy, the aggregates were identified as ordered protein filaments that required lanthanum to polymerize. These filaments could be disassembled by the addition of a chelating agent. A simple head-to-tail model is proposed for filament formation that explains the metal ion-dependency. The model is supported by the capping of one of the polypeptides with biotin, which disrupts filament formation and provides the ability to control the average length of the filaments.

**Conclusion:**

Metal ion-dependent, reversible protein filament formation is demonstrated for two designed polypeptides. The polypeptides form filaments that are approximately 3 nm in diameter and several hundred nm in length. They are not amyloid-like in nature as demonstrated by their behaviour in the presence of congo red and thioflavin T. A capping strategy allows for the control of filament length and for potential applications including the "decoration" of a protein filament with various functional moieties.

## Background

**Figure 1:**
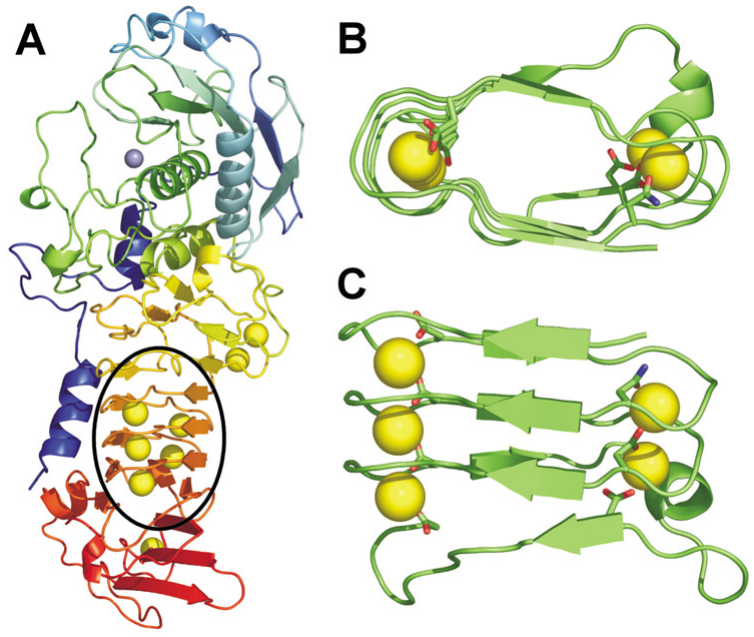
**The crystal structure of alkaline protease from *Pseudomonas aeruginosa *(1KAP) to 1.64 Å and the β-roll domain in isolation**. **A) **Full structure of the zinc metalloprotease [7] showing the zinc atom coordinated in the active site (blue sphere) and the calcium binding β-roll circled in black; **B) **Enlarged view looking down the axis of the β-roll of alkaline protease including the five bound calcium ions (orange spheres), the parallel β-strands and tight turns around the metal-binding sites and the side chains of the seven residues making contact with Ca^2+ ^ions: Asp338, Asn347, Asp356, Asp365, Asp374, Asp 390 and Asp400; **C) **Enlarged view of the front face of the β-roll of alkaline protease with the same residues highlighted as in panel B.

There are many types of protein folding motifs formed by simple repetitive sequences, or so-called solenoid proteins [1]. One example is the β-roll motif, which at its most basic, is a repeating unit of a short β-strand linked to a second short beta-strand by a turn region comprising two approximately 90 degree bends in the polypeptide backbone. This unit then repeats as another turn region links further β-strands into the unit forming a stack of parallel β-sheets, one on either side of the β-roll motif. An example of such a motif from the X-ray structure of *Pseudomonas aeruginosa *alkaline protease is presented in Figure 1. The simple relationship between sequence and protein structure of solenoid proteins including β-roll, β-helical, leucine-rich repeat and ankyrin repeat proteins has been well documented previously [1]. β-roll containing protein folds have also been reviewed previously so only a short introduction is given here [2,3].

It has also previously been put forward that parallel β-strands and β-helical or β-roll repeating motifs are found in many amyloid filaments and fibrils [2]. To date, the β-roll model of HET-s fibrils from *Podospora anserina *[4] is the most plausible β-roll-like model of an amyloid filament and has been supported by recent electron miscroscopy studies [5] in addition to mutational analysis of HET-s derived protein sequences.

β-roll domains have been found in a number of proteins, often as a subdomain that binds divalent metal ions (Ca^2+^), as in the "zincin" family of metalloproteases [6-8]. including alkaline protease from *Pseudomonas aeruginosa*, see Figure 1 to indicate the position of the β-roll domain within the protease[7]. This β-roll is composed of glycine- and aspartate-rich nonapeptide repeats with a GGXG(X/D)DXUX consensus sequence (where G = glycine, X = any amino acid, D = aspartate, and U = a large hydrophobic residue such as Leu, Ile, Val, Phe, Tyr) [7].

Similar nonapeptide repeats are present in the C-terminal regions of the 100 – 200 kDa repeat-in-toxin (RTX) family of cytotoxic and hemolytic toxins from Gram negative bacteria [9-15] and the I.3 family of lipases [16,17]. In addition, a right-handed β-roll domain containing nonapeptide repeats has been structurally characterized in the R-module of the AlgE4 C-5 epimerase from *Azotobacter vinelandii *by nuclear magnetic resonance (NMR) [18]. Structural and experimental data suggest that calcium ions can be bound within the turns of the R-module β-roll domain to stabilize the overall protein.

The RTX metal-binding nonapeptide repeat has been the subject of two previous studies that exploit the change in conformation of the motif upon calcium binding. A study by Lilie *et al*. used a synthetic 75-residue polypeptide (NH_2_-WLS [GGSGNDNLS]_8_-COOH) as a minimized model of the β-roll domains from RTX toxins [19]. The synthetic β-roll irreversibly bound calcium in the millimolar range and showed a change in conformation in the presence of 100 mM CaCl_2 _and 25% PEG 8000 as monitored by far-UV circular dichroism (CD). The minimized β-roll behaved similarly to the β-roll regions of *B. pertusis *adenylate cyclase toxin and *E. coli *hemolysin. The early success of minimizing the β-roll domain was followed by a study using the GGXGXDXUX nonapeptide repeat β-roll motif from *Serratia marcescens *serralysin as a calcium switching "molecular spring" fused between two 6-phospho-β-galactosidase (PGAL) monomers [20]. In the presence of calcium, the β-roll formed a 2 nm long rod linking the two PGAL units, similar to a molecular dumbbell when viewed by electron microscopy (EM). Upon addition of ethylenediaminetetraacetic acid (EDTA) the β-roll adopted an extended conformation and the two PGAL units became more distant from one another as demonstrated by EM.

**Figure 2:**
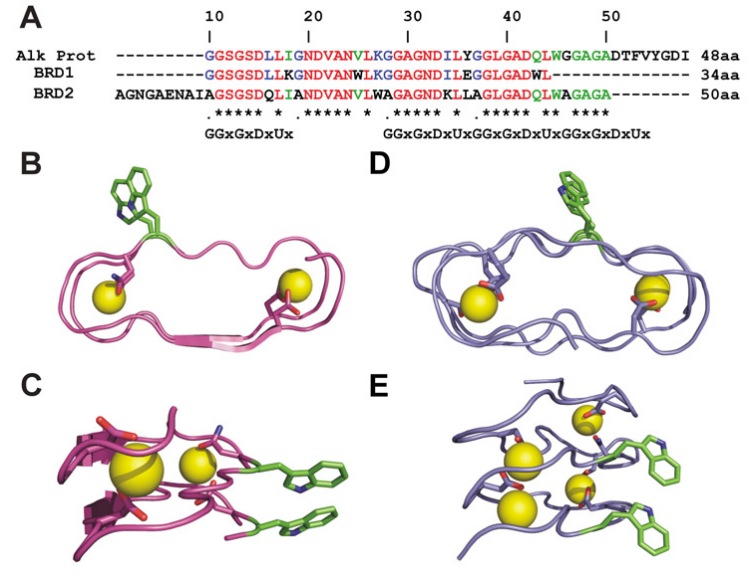
**The amino acid sequence and modeled structures of BRD1 and BRD2**. **A) **Amino acid sequence alignment of the β-roll from *P. aeruginosa *alkaline protease with BRD1 and BRD2, highlighting the GGxGxDxUx repeat. Identical residues are indicated by an asterisk; **B) **The modeled structure of BRD1 highlighting the pair of tryptophan residues and two calcium-binding sites composed of residues Asp6, Asn15, Asp24 and Asp33; **C) **The back face of the BRD1 model showing the stacked Trp pair in more detail; **D) **The modeled structure of BRD2 highlighting the pair of tryptophan residues and four calcium-binding sites composed of residues Glu6, Asp15, Asp 21, Asn24 and Asp33 and Asp 42; **E) **The back face of the BRD2 model showing the stacked Trp pair in more detail.

The success of the above β-roll models inspired our design of two Beta-Roll Designed polypeptides (BRD1 and 2) based on the nonapeptide consensus sequence from alkaline protease [9]. An alignment of the amino acid sequences of alkaline protease, BRD1 and BRD2 is presented in Figure 2A. In the X-ray structure of alkaline protease from *P. aeruginosa *[7] there are a total of seven nonapeptide repeats that together bind five Ca^2+ ^ions to form a right-handed β-roll with three parallel β-strands on each side of the motif (Figure 1). The repeats form short β-strands (4–5 residues) linked by tight turns where aspartate side chains, peptide backbone carbonyl groups and water molecules bind Ca^2+ ^ions internally to stabilize the motif. The Ca^2+ ^ions are generally sandwiched between two neighbouring turn regions which each contribute a side chain carbonyl group to the coordination. In addition, three to five backbone carbonyl groups, and one or two water molecules complete a six-coordinate (approximately octahedral) binding site.

BRD 1 was designed as a 34-amino-acid minimized β-roll motif containing three β-strands and two calcium-binding sites, see Figure 2B and 2C. BRD1 was intended to be stabilized by the coordination of two metal ions and the introduction of a pair of stacked tryptophan residues (W16 and W34), which have previously been shown to stabilize short, soluble, β-hairpin proteins [21]. Residues 9 (I to K) and 27 (Y to E) of BRD1 were altered from the alkaline protease sequence to introduce a potential salt bridge between β-strands. BRD2 is an elongated version of BRD1 (50 amino acids) that is comprised of five β-strands with four calcium-binding sites, and also includes a stacked pair of tryptophan residues (W27 and W45) as shown in Figure 2D and 2E. Once again charged residues were introduced to the sequence (L to Q at position 16 and I to K at position 34) to promote stability and solubility. The N-terminal extension contains a modified nonapeptide repeat with asparagine side chains to potentially bind calcium and a glutamic acid residue to add further charged character to the polypeptide. Several glycine residues were replaced with alanine to limit the flexibility of the BRD2 polypeptide. The folded BRD2 motif is approximately 30 Å wide, 25 Å deep and 15 Å high.

The BRD polypeptides were originally designed to fold upon the addition of calcium and form soluble monomeric β-rolls that could be used as scaffolds for the protein engineering of various functions including ice binding mimicking insect antifreeze proteins. However, the data presented below demonstrate that the BRD polypeptides undergo ordered and reversible metal ion-dependent protein polymerization in the presence of lanthanum ions. The process generates very long, thin, unbranched filaments that exhibit similarities and differences to classical amyloid fibrils.

## Results

### La^3+ ^causes ordered aggregation of the BRD polypeptides

**Figure 3:**
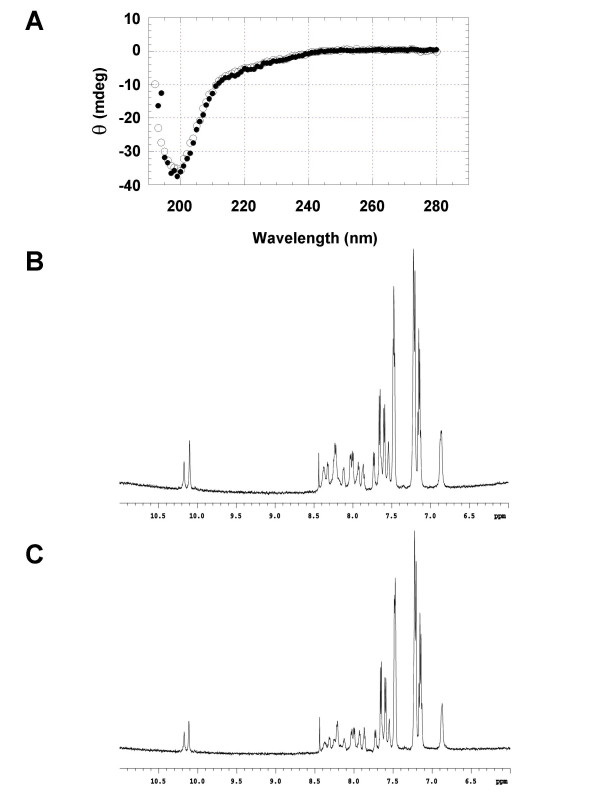
**Circular dichroism and 1D NMR data for BRD1 and 2**. **A) **Circular dichroism spectra for a solution of BRD2 with (white circles) and without (black circles) 10 mM CaCl_2 _at room temperature. **B) **1D NMR data for ~0.5 mM BRD1 in 90% H_2_O,10% D_2_O at pH 6.5. **C) **1D NMR data for BRD1 as above plus 3.85 mM CaCl_2_. This figure shows the aromatic, amide NH, and indole NH region (6.0 to 11.0 ppm) of 600 MHz ^1^H NMR spectra.

Initial analysis by CD and 1-D NMR showed no obvious conformational change upon the addition of calcium to the BRD polypeptides as shown in Figure 3. It is clear that the addition of 10 mM CaCl_2 _to BRD2 did not change the conformation of the polypeptide. The CD spectra of BRD2 in Figure 3A are indicative of random coil structure, suggesting that the BRD polypeptides are unstructured even in the presence of calcium. Figures 3B and 3C show 1D NMR data for BRD1 in the presence and absence of CaCl_2 _respectively. The spectra are indicative of an unstructured peptide, as judged by the narrow line widths and the lack of spectral dispersion from the expected chemical shifts of a free peptide. No change was observed as calcium was added, beyond a small amount of broadening at the highest calcium concentrations (~100 mM) indicating possible aggregation. The intrinsic fluorescence of the stacked tryptophan pairs was also analyzed and suggested only a slight change towards a more hydrophobic environment upon the addition of calcium (not shown), further evidence that the polypeptides were not subject to a significant conformational change upon the addition of metal ions.

The trivalent lanthanide metal lanthanum was substituted for calcium. It has a very similar ionic radius but an additional charge which enables the ion to occupy calcium-binding sites with a higher affinity than calcium [22]. The addition of lanthanum chloride to a solution containing either BRD polypeptide produced a cloudy suspension rendering CD, NMR and fluorescence experiments ineffective.

**Figure 4:**
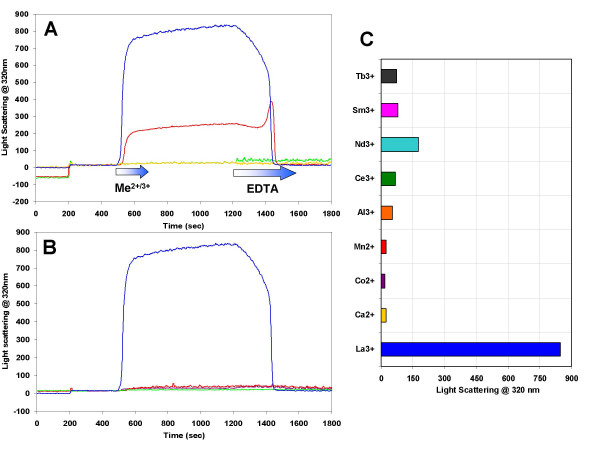
**Right angle light scattering timedrive plots for BRD1, 2 and control peptides/proteins in the presence of calcium or lanthanum and the maximum right angle light scattering responses using a variety of metal ions**. The proteins were added at 200 s and the metals were introduced using a syringe pump starting at 500 s for a total of 120 s. A solution of EDTA was added to the cuvette using a syringe pump beginning at 1200 s until the right angle light scattering signal fell to its original level. **A) **Timedrive data for BRD1 with calcium (yellow line), BRD1 with lanthanum (red line), BRD2 with calcium (green line) and BRD2 with lanthanum (blue line). This plot illustrates that an increase in right angle light scattering (and therefore filament formation) is specific for peptides in solutions containing lanthanum and that it is a reversible process. This plot also highlights the larger increase in scattering for BRD2 over BRD1 at identical concentrations. **B) **Timedrives for a protein free control with lanthanum (purple line), a 15-mer peptide with α-helical propensity (red line), CAST (green line) and BRD2 with lanthanum (blue line) highlighting that the increase in right angle light scattering is specific to the BRD polypeptides only. **C) **A bar chart displaying maximum right angle light scattering responses at 320 nm for various metals at 10 mM final concentration in solutions containing 100 μM BRD2 peptide.

The aggregation of the BRD polypeptides was more closely analyzed using right angle light scattering timedrive experiments. A summary of the initial experiments illustrating the lanthanum dependence, BRD polypeptide specificity and reversibility of the aggregation event is presented in Figure 4. A very large increase in right angle light scattering (and therefore particle size in the solution) is produced by both BRD1 and 2 polypeptides upon addition of lanthanum (blue and red lines in Figure 4A). The increase in right angle light scattering occurs at around 10–20 s after the initiation of lanthanum addition (at 500 s) for both BRD polypeptides. The signal is 4-fold higher for the larger peptide. Calcium addition to solutions of BRD1 and 2 (green and yellow lines in Figure 4A) does not increase right angle light scattering. This suggests that calcium ions are not capable of initiating an aggregation event. After the lanthanum addition is stopped (at 620 s) the right angle light scattering signal remains fairly constant although it does have a slight positive slope suggesting continued increase in particle size with time. The addition of EDTA to suspensions of BRD1 or 2 aggregates at 1200 s immediately halts the gradual increase in right angle light scattering, after which the loss of right angle light scattering is accelerated as more EDTA is introduced into the cuvette (red and blue lines in Figure 4A). This suggests that the increase in particle size is caused directly by lanthanum and can be reversed by chelating the La^3+ ^ions. The sharp increase then decrease in scattering at 1450 s exhibited by BRD1 (red line in Figure 4A) was a generally observed characteristic of the BRD 1 peptide but was not observed for BRD2 suspensions. The marked increase in scattering exhibited by the larger BRD2 peptide relative to BRD1 at a similar concentration was also consistently seen.

The addition of lanthanum to other unrelated peptides and proteins did not produce a significant increase in right angle light scattering, suggesting that aggregation of peptides in the presence of lanthanum is not a general phenomenon (Figure 4B). The controls included a 15-residue peptide corresponding to the N-terminal sequence of m-calpain (a cysteine protease) large subunit [23] (1578.9 Da) (red line in Figure 4B) and calpastatin (CAST), a relatively unstructured 10.5-kDa protein that specifically inhibits calpain [24] (green line in Figure 4B). The final experiment in Figure 4B shows that a peptide-free control (purple line in Figure 4B) did not produce an increase in right angle light scattering thereby confirming that the observed increases for the BRD polypeptides must be the result of specific peptide-La^3+ ^ion interactions. The addition of EDTA to these control solutions has no effect on the scattering signal. Figure 4C highlights the specificity for lanthanum (blue bar) as other divalent and trivalent metal ions failed to produce large increases in right angle light scattering as a result of protein-metal aggregation.

### BRD1 and 2 aggregates are protein filaments

**Figure 5:**
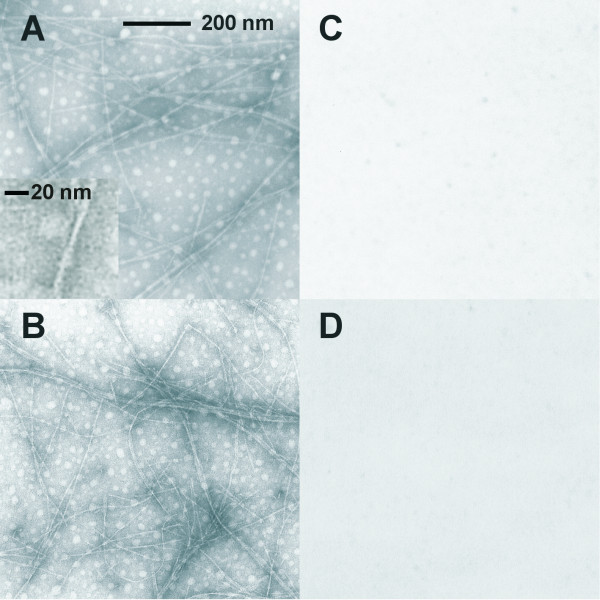
**Negative-stained EM images of BRD1 and 2 filaments at 30,000 × magnification on Formvar-coated nickel grids**. **A) **BRD1 filaments in a lanthanum solution; **B) **BRD2 in a lanthanum solution; **C) **BRD2 in a calcium solution; **D) **BRD2 in a lanthanum solution with EDTA added post filament formation. The images clearly show that filaments are only formed by BRD polypeptides in the presence of lanthanum and that they can be disassembled by the addition of EDTA.

Four EM images of BRD1 and 2 peptide filaments and controls are presented in Figure 5. It is clear from Figure 5A and 5B that smooth peptide filaments are formed by BRD1 and 2 in the presence of La^3+ ^ions. Bundles of filaments, some showing a tangled topology, can also be seen. The unbranched and flexible nature of the filaments is readily apparent. From these EM images it is possible to estimate the dimensions of the filaments. They vary in length from only a few hundred nanometers to a few micrometers, although the exact length is hard to accurately gauge as the filaments overlap each other in long bundles. The diameter of a single filament appears to be around 3 nm, though they can be seen to clump and form bundles that are up to 40 nm thick. The general diameter of the BRD filaments is similar to that of amyloid protofibrils associated with neurodegenerative diseases. However, the appearance of the BRD1 and 2 filament bundles are less ordered and much thinner than that of amyloid fibrils [25].

Figure 5C shows an EM grid that was prepared from a solution of BRD2 with calcium ions in place of lanthanum ions and no protein filaments are observed. This confirms that calcium is not capable of causing the BRD polypeptides to aggregate into filaments. Figure 5D is an example of a grid that was prepared from a suspension of BR2-La^3+ ^filaments but was subsequently treated *in situ *with a solution of EDTA. The filaments that were previously visible have disassociated and are no longer visible by negative staining. This finding supports the right angle light scattering observations of EDTA causing filament dissociation by removing lanthanum ions from the aggregates.

### Modelling suggests the BRD filaments are head-to-tail polymers

**Figure 6:**
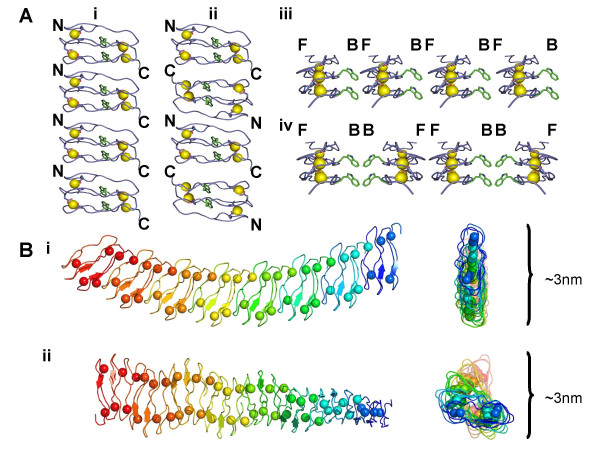
**Schematic representations of how BRD2 monomers could polymerise as long thin filaments**. Key: N = N terminus, C = C terminus, F = Front Face, B = Back Face. **A) **The models are **i) **head-to-tail; **ii) **head-to-head and tail-to-tail; **iii) **front face to back face; **iv) **front face to front face and back face to back face. The favoured model for formation of very long filaments is the head-to-tail formation. **B) **Models of docked BRD2 monomers that are of the correct dimensions to form 3 nm diameter filaments that could extend over hundreds of nm. **i) **head-to-tail BRD2 monomers with their front faces all aligned on one side of the filament creating a bent polymer; **ii) **head-to-tail BRD2 monomers with their front faces on alternating sides of the filament creating a twisted polymer.

If filaments that are several hundred nanometers long can form, then the interaction between the monomeric units must be repetitive. Considering the BRD2 peptide, one obvious area for an interaction between monomers is between termini to linearly extend the filament. Interactions between the termini could be of a head-to-tail (Figure 6Ai) or head-to-head variety (Figure 6Aii). The most likely interaction in a very long polymer would be head-to-tail as this unit could be repeated indefinitely. A head-to-head interaction would also require a second, distinct, tail-to-tail interaction, Figure 6Aii. If the interaction is between the faces of the BRD polypeptide, a similar case would occur where there could be a front-to-back interaction that repeats endlessly, see Figure 6Aiii. There could also be a front-to-front interaction in addition to a back-to-back interaction but this would position four tryptophan residues in close proximity which is likely to be unfavourable, as shown in Figure 6Aiv.

Metal ions must also play a part in the interaction and there are already four designed metal-binding sites in the turn regions of the monomeric BRD polypeptides. It is possible that extra lanthanum ions bind between two half metal binding sites, composed of the Asp and Asn carbonyl groups not already bound to a metal ions and peptide backbone carbonyls, found in the N and C termini of different monomers, essentially gluing two monomers together in a head-to-tail fashion. There are two Asn side chains and one Glu side chain close to the turn regions in BRD1 and two Asp residues in BRD2 that could form half metal binding sites near the two termini of the monomers. It is harder to imagine metal ions binding between faces of BRD monomers as the only carbonyl donors in this region would be from peptide side chains. The only external carbonyl containing side chains in BRD1 are close to the turns as previously mentioned. There are no external Asp or Glu side chains and only two external Asn side chains on BRD2, and these are both near the first N-terminal turn region.

Our prediction is that the BRD monomers interact via a metal-dependent head-to-tail interaction between the N and C termini of two different monomers. This is the simplest repeating unit that could form very long polymers. In addition, the width of such a repeating unit would be around 3 nm, the width of a single filament measured from EM images. Two models are proposed for the polymerization of BRD2 into a protein filament, as shown in Figure 6B. The first is a head-to-tail repeating unit where the front and back faces of BRD2 are always on the same side which produces a bend in the polymer. The second model is based on the front and back faces of the BRD monomer alternating, which causes a twist. These topologies have been observed by EM. Similar models would also be feasible for BRD1 polymerization.

### Testing the filament model with a capping modification

**Figure 7:**
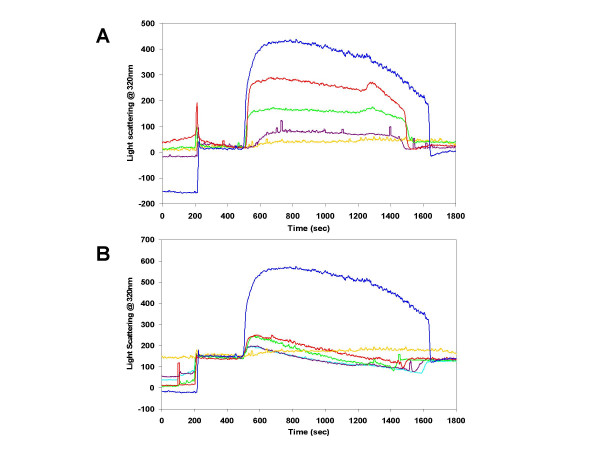
**Right angle light scattering timedrive plots for BRD2, BioBRD2 and mixtures of the two peptides in the presence of lanthanum. **The proteins were added at 100 (BRD2) and 200 s (BioBRD2) and the metals were introduced using a syringe pump starting at 500 s for a total of 120 s. A solution of EDTA was added to the cuvette using a syringe pump beginning at 1200 s until the right angle light scattering signal fell to its original level. **A) **Timedrive illustrating the relative right angle light scattering responses of 10 μM BRD2 (blue line) 10 μM (yellow line), 20 μM (purple line), 30 μM (green line) and 60 μM (red line) BioBRD2. It is clear that BioBRD2 does not produce increases in right angle light scattering comparable to BRD2, suggesting it does not form stable filaments. **B) **Timedrive illustrating the relative right angle light scattering responses of 10 μM BRD2 (blue line) BioBRD2 alone (yellow line), a 1 to 1 (cyan line), 1.5 to 1 (purple line), 4 to 1 (green line) and 9 to 1 (red line) excess of unmodified BRD2.

The simple end-to-end repeating model was tested by synthesizing a modified BRD2 polypeptide that was tagged with biotin at the N-terminus (BioBRD2). Right angle light scattering timedrives using BioBRD2 compared to unmodified BRD2 are presented in Figure 7A. The increase in scattering response for 10 μM BioBRD2 (yellow line) was 10% of the response observed for 10 μM BRD2 at the same concentration (blue line). This immediately suggested that the modified BRD2 polypeptide could not polymerize as well as the unmodified peptide. Samples of 20 μM (purple line), 30 μM (green line) and 60 μM BioBRD2 (red line) were also analyzed and a steady increase in scattering was observed. However, even after a 6-fold increase in concentration the maximum observed response for BioBRD2 (red line) was only two thirds of that for 10 μM BRD2 (blue line). Once again, the aggregation causing an increase in right angle light scattering was reversible upon addition of EDTA as shown by the drop in signal beginning at 1200 s and a return to baseline levels by 1650 s. However, less EDTA was required to reduce scattering to the pre-La^3+ ^baseline for the BioBRD2 samples than for BRD2. Scattering signals were reduced to baseline levels after 1500 s for the 10–60 μM BioBRD2 samples (yellow, purple, green and red lines, respectively).

The addition of BioBRD2 to BRD2 severely disrupts filament formation (Figure 7B). Samples all contained a total peptide concentration of 10 μM but were comprised of a mixture of BioBRD2 and BRD2 peptides. Samples contained a 1 to 1 (cyan line), 1.5 to 1 (purple line), 4 to 1 (green line) and 9 to 1 (red line) molar excess of unmodified BRD2. These samples were compared to the BRD2 (blue line) and BioBRD2 (yellow line) data from Figure 7A. Hardly any increase in scattering was observed with the 1 to 1 mixture of BRD2 to BioBRD2 (cyan line) while even at 9 to 1 BRD2 to BioBRD2 (red line) the maximum scattering increase was only 24% of that for the same total concentration of BRD2 alone (blue line). Intermediate ratios of the BRD polypeptides showed a slight increase in scattering in proportion to the amount of BRD2 present but the increase in scattering was unstable as shown by the negative slope for each of the datasets.

**Figure 8:**
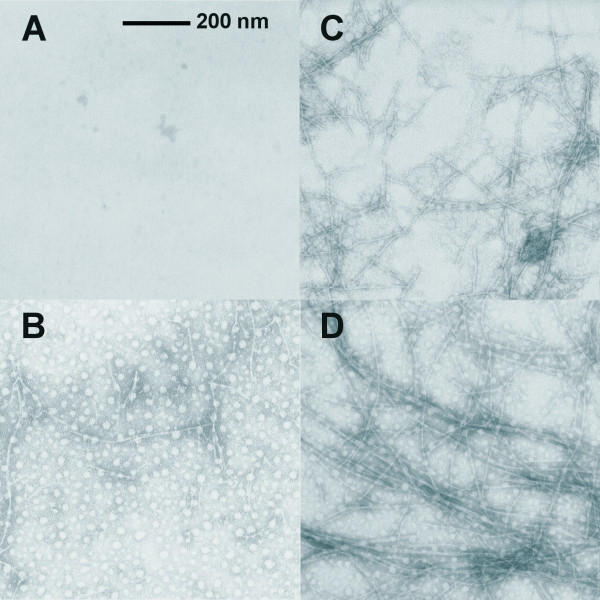
**Negative stained EM images of BioBRD2 and BRD2 in lanthanum solution**. **A) **BioBRD2 alone; **B**) 2:1 mixture of BRD2:BioBRD2; **C**) 20:1 mixture of BRD2:BioBRD 2; **D**) 40:1 mixture of BRD2:BioBRD2. These images at 30,000 × magnification on Formvar-coated nickel grids show the inverse relationship between the concentration of biotinylated BRD2 and the length of filaments formed. BioBRD2 does not form filaments in lanthanum solutions and it reduces the length and abundance of BRD2 filaments compared to identical conditions with BRD2 alone.

Further evidence for the disruption of filament formation by modifying the N-terminus of BRD2 was provided by EM images of samples containing a range of BRD2 to BioBRD2 ratios in the presence of lanthanum, which are summarized in Figure 8. BioBRD2 in lanthanum (Figure 8A) produced no visible filaments while the 2 to 1 mixture of BRD2 to BioBRD2 (Figure 8B) produced only a few short filaments. Increasing the ratio to 5 to 1, 10 to 1 and 20 to 1 produced an increase in the quantity and length of filaments observed (See Figure 8C for the 20:1 EM image). A 40 to 1 excess of BRD2 over BioBRD2 produced filaments that were similar in appearance and almost as long and abundant as conditions containing only unmodified BRD2 (Figure 8D and Figure 5B).

### Controlling filament length by capping

**Figure 9:**
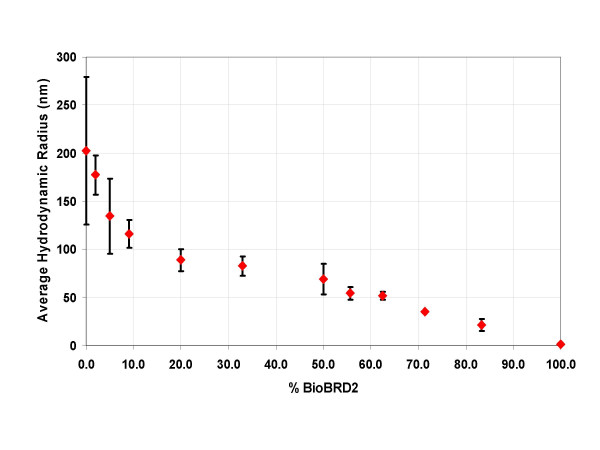
**Plot of the average hydrodynamic radius (nm) against the amount of BioBRD2 (%) compared to unmodified BRD2. **A 100% BioBRD2 sample represents a solution free of BRD2 while a 50% BioBRD2 solution corresponds to a 1 to 1 mixture of the two peptides. Y-axis error bars represent the standard deviation between three samples for each data point. Note the inverse correlation between increase in hydrodynamic radius and decreasing amount of BioBRD2.

Data collected by right angle light scattering and EM suggested that the length and abundance of the BRD filaments could be controlled by varying the amount of BioBRD2 relative to unmodified BRD2. To test this, dynamic right angle light scattering (DLS) was employed to analyze the relationship between particle size and the ratio of BRD2 to BioBRD2. A range of solutions was tested in triplicate and the average hydrodynamic radius plotted against the amount of BioBRD2 in the solution as shown in Figure 9. The inverse relationship between hydrodynamic radius (and thus particle size) and the amount of BioBRD2, is pronounced and appears to have two phases. The initial phase is a linear increase in particle size between 100% and 50% BioBRD2. Between 50% and 20% BioBRD2 there is little change in hydrodynamic radius suggesting an equilibrium has been obtained. Below 50% BioBRD2, the increase in hydrodynamic radius appears to be more geometric in nature and the radius is also more variable as displayed by the larger error bars nearer 0% BioBRD2. DLS data reiterate the right angle light scattering and EM findings that altering the N terminus of BRD2 can lead to a method for capping the filaments, thereby controlling filament length and abundance. This supports our simple model of filament formation by repeating head-to-tail N to C terminus interactions.

### La^3+ ^ions are present in excess over the BRD polypeptides

Amino acid analysis data are summarized in Table 1 and highlight the close match between experimental and expected composition of the peptides. Only nine amino acids are present in appreciable amounts, of which Asx, Ala, Gly and Leu predominate. There are only trace amounts of amino acids like Tyr, Phe, Met that do not appear in the sequence; and Trp, although present in the sequence, is missing because it is not stable to the conditions used for acid hydrolysis of the peptide. These data also prove that the filaments are proteinaceous in nature. Amino acid analysis data also allowed an estimation of the amount of peptide that aggregates. Compared to values for the starting solution of BRD2, the pellet and the remaining supernatant, around 75% of the peptide formed aggregates. Elemental analysis of the same samples for La^3+ ^suggested that the pellet contained approximately 50% of the added lanthanum giving a ratio of La^3+ ^to BRD polypeptide of 75 to 1. This is far from the expected 4 to 1 ratio. Calculations from the right angle light scattering data suggest that around 1.0–1.5 μmoles of lanthanum are added to the cuvette containing 0.03 μmoles of BRD polypeptide. This produces a minimum ratio of 33 to 1 and a maximum ratio of 50 to 1 La^3+ ^ions to BRD polypeptide. Attempts were made to wash precipitated fibrils to remove unbound or non-specifically bound La^3+^. Following resuspension in water and centrifugation, the BRD2 filament pellet decreased in size suggesting a removal of weakly bound lanthanum ions (possibly between BRD2 monomers) leads to some degree of filament dissociation. Unfortunately, elemental analysis of these wash fractions yielded concentrations below the detectable limit of La^3+ ^by ICP-AEOS (less than 0.1 ppm or μM concentrations).

**Table 1:** Amino acid analysis data for BRD2 protein filaments

**A) Amino Acid**	**B) Picomoles in Sample**	**C) % of Total**	**D) Expected % in BRD2 Seq**
**Asx**	594.2	14.6	18
**Glx**	222.1	5.8	6
**Ser**	152.9	4.0	4
**Gly**	844.0	22.0	20
**Ala**	1022.0	26.5	26
**Val**	199.0	5.2	4
**Ile**	152.1	3.9	4
**Leu**	588.0	15.2	12
**Lys**	82.1	2.1	2
	
	**3856.4**	**100**	**96**

**A) **Amino acids listed are those for which a signal above background was measured. Asx is the combination of Asp and Asn, Glx is the combination of Gln and Glu. **B) **Amount of amino acid present in pmols. **C) **Amount of amino acid expressed as a percentage of the sum. **D) **Amount of amino acid expected based on sequence of BRD2. Two tryptophan residues in the BRD2 peptide were destroyed during the acid hydrolysis and derivatization of the resulting amino acids prior to amino acid analysis, which accounts for the remaining 4 % of the total picomoles of sample.

### The BRD filaments are not amyloid fibrils

Finally, the BRD filaments were analyzed for their similarity to amyloid fibrils. Two standard spectrometric assays were used, thioflavin T binding [26] and congo red binding [27]. BRD2 filaments had much lower fluorescent emission at 482 nm (3.0 × 10^4 ^AFU) compared to a sample of Aβ40 amyloid fibrils (1.95 × 10^5 ^AFU) in the presence of thioflavin T. In addition, BRD2 filaments did not produce a characteristic spectral shift towards 550 nm upon congo red binding compared to serum amyloid A fibrils (data not shown). The A_540nm_/A_480nm _for BRD2 was 0.045 compared to 0.16 for serum amyloid A fibrils. Control experiments using Aβ-40 in the presence and absence of 10 mM LaCl_3 _demonstrated that lanthanum had no intrinsic affect on amyloid fibril staining using thioflavin T and congo red (data not shown).

## Discussion

It is clear that these BRD polypeptides have some very interesting and unusual properties. In the presence of La^3+ ^they form long, thin, unbranched filaments with some tendency to associate side by side to form bundles up to 40 nm thick and/or become tangled. We propose a model for these based on a simple head-to-tail repeating unit of BRD monomers that interact via the N- and C-terminal regions with lanthanum ions bound to the designed metal-binding sites. Lanthanum ions may also interact with charged side chains between the N and C termini of two different monomers, effectively linking the BRD monomers together. This model allows for the polymerization of the BRD monomer by the formation of very long continuous parallel β-sheets. These long β-sheets can then interact with more lanthanum ions via charged side chains and form bridged interactions with other β-roll filaments, allowing for the formation of filament bundles. Branched filaments would be unlikely to form if this model holds true as the head-to-tail interaction does not allow for two BRD monomers to interact at the C terminus of a single monomer. Experimental data support this suggestion as branched filaments have not been observed by EM.

The addition of EDTA causes the filaments to readily disassociate and dissolve. This is a novel feature as peptide aggregates are often very hard to resolubilize. Amino acid analysis proved that the filaments are proteinaceous, with a profile that matches the composition of the BRD polypeptides. Therefore, the observed increase in right angle light scattering is not simply due to a precipitate of insoluble lanthanum compounds. One explanation for disassembly of the filaments in EDTA would be removal of La^3+ ^from the monomer interface thereby destabilizing the structure of the building block. If this happened randomly along the filament the internal breaks would quickly cause filament disassembly. The exact amount of EDTA required to disassociate the β-roll filaments is unclear but based on right angle light scattering timedrive data in Figure 4, EDTA begins to have an effect relatively early in its introduction to the sample (observed as a decrease in right angle light scattering) but it takes around 240 s for the signal to return to baseline. This is twice the length of the lanthanum injection time course although the lanthanum stock is 1 M whereas the EDTA stock is 0.5 M which suggests an equal amount of EDTA is required. Presumably, EDTA would first bind any free La^3+ ^then begin to remove La^3+ ^ions from the filaments leading to disassociation. The dissolution of some of the BRD filaments under washing conditions suggests that a portion of La^3+ ^is bound weakly, most likely the ions bound between BRD monomers and any non-specifically bound ions. The stability constant (K_stab_) of the La^3+^-EDTA complex is 5.01 × 10^15 ^Mol^-1 ^[28], and as the dissociation constant (K_d_) = 1/K_stab_, the K_d _for La^3+^-EDTA is 2.0 × 10^-16 ^Mol^-1^. This binding is 100,000 times tighter than that between EDTA and Ca^2+^. EDTA therefore has such a strong affinity for La^3+ ^ions that it will likely outcompete the affinity of La^3+ ^for the designed metal-binding sites in the BRD polypeptides. Even if the lanthanum were bound tightly to the polypeptide, EDTA would be able to form stable complexes and lead to dissociation of the BRD filaments.

The BRD filaments only form in the presence of peptide and lanthanum ions. Several controls where carried out using divalent and trivalent metal ions (Ca^2+^, Mg^2+^, Mn^2+^, and Al^3+^) and none of these metals gave an increase in right angle light scattering due to filament formation, as shown in Figure 4C. Other lanthanoid metals were tested (Ce^3+^, Nd^3+^, Sm^3+ ^and Tb^3+^) and some of these gave small right angle light scattering increases. However, no filaments were visible by EM (data not shown). It is interesting to note that studies on the R-module of AlgE4 from *Azotobactor vinelandii*, where the lanthanide thulium was used in place of calcium, gave a precipitate at 6 mM or higher thulium [18]. Thulium ions were also shown to be binding to negative residues on the surface of the R-module and in the unordered C-terminal region. The precipitate was not analyzed further so it is not known if protein filaments formed.

Amino acid analysis and elemental analysis data suggest that around 75 La^3+ ^ions are present for every BRD monomer and not all of the peptide and metal present in solution forms filamentous aggregates. A slightly lower ratio was obtained from right angle light scattering data but lanthanum is clearly in excess and therefore cannot simply be binding just to the four designed calcium-binding sites in the BRD polypeptides. Analysis of stained but unwashed EM grids showed a huge amount of electron density which was easily washed off by rinsing the grids in water before placing them on the EM. This may be excess lanthanum that precipitates with the filaments, artificially raising the amount of lanthanum associated with the peptide filaments.

The BRD filaments are similar in diameter and appearance to some amyloid protofibrils [25] but do not always form highly ordered bundles. The bundles that do form are not as thick as those seen for amyloid under EM and atomic force microscopy (AFM) [25,29]. There is also variability in the length of the filaments. Filament length increases as a function of time; longer growth times produce longer fibrils. On a short time scale, the scattering of samples in the presence of lanthanum would slowly increase until the addition of EDTA was initiated, suggesting that the filaments were increasing in size. On a longer time scale, samples that were set up for EM showed a general increase in filament length the longer they were incubated on the EM grids prior to staining. This suggests the filaments are growing from a nucleus and additional BRD monomers are added to the ends of the filament nucleus. In addition, the scattering responses of BRD2 polypeptides were larger than those seen for BRD1 polypeptides at an identical concentration. The BRD2 polypeptide is 16 amino acid longer (~50% larger) than BRD1 and 1.3 kDa larger in mass. The difference in size and mass between these two polypeptides would be magnified in a filament where the units repeat and could explain the higher scattering responses for BRD2 filaments. The models we present in Figure 6 fit the experimental data and the continual addition of further BRD polypeptides via N- to C-terminal interactions could produce filaments that are hundreds of nanometers in length. The model assumes the peptides do adopt a β-roll-like conformation as the diameter of a single filament (~3 nm) closely matches that of the β-roll peptide model (3 nm) in Figure 2.

The modification of BRD2 with an N-terminal biotin residue lends further support to our model of filament formation. The modified peptide can clearly inhibit filament formation and does not form filaments alone. Therefore, the N terminus of the BRD peptide is important in the aggregation process. The ability to exert some control over filament length and abundance by altering the amount of BioBRD2 is of great interest and practical application. This capping strategy could be altered to have other modified BRD polypeptides with biotin or other functional groups positioned elsewhere on the BRD unit which would allow for decoration of the filaments with other moieties such as enzyme subunits, fluorescent tags or antibodies. The degree of decoration could be controlled in an analogous manner to the capping strategy. A higher proportion of modified BRD monomers would lead to more decoration and *vice versa*. In theory it is possible that a filament could be grown with a core of BRD2 peptides, an N-terminal cap and several different modified BRD polypeptides. Protein and peptide-based nanomaterials are of great interest and have many potential applications, several of which have been reported recently [30-34].

The aggregation of β-sheet rich proteins is not uncommon as β-strands are intrinsically insoluble [35] and nature has evolved several ways in which to avoid the aggregation of proteins containing β-sheets [36]. The *de novo *design of soluble β-sheet proteins has proven to be particularly challenging but there have been some notable successes [21,37-39]. With the challenges of designing soluble β-sheet proteins in mind, it is unsurprising that many designed β-sheet proteins form aggregates (either by misfolding or implicit design) and some of these are fibrillar in nature [40-43]. It is also possible to convert a protein that would normally from ordered aggregates into a soluble monomeric from [44] or assembly can be blocked by the addition of various inhibitors [45-47]. Our BRD polypeptides are another example of a designed protein that forms an ordered aggregate. However, they can be disassembled simply by the addition of chelating agents such as EDTA and the addition of a biotin residue at the N-terminus can block filament polymerization.

In-register parallel β-sheet arrangements have been demonstrated for several proteins [48-51]. However, these folds do not closely resemble β-rolls as the β-strands of a β-roll are not in-register. Prion protein fibrils have been modelled as β-helical repeating units (similar to β-rolls but the turn regions are less tight) but this model does not stand up to scrutiny as well as the spiral model of prion protein fibrils [52]. Similarly, Sup35 fibrils have also been modelled as β-helices with parallel β-strands similar to those found in β-rolls [53] but the model has been invalidated [54]. The β-roll model of HET-s fibrils from *Podospora anserina *[4] is the most similar model but does not rely on metal-binding and is not reversible. However, there is evidence that metal ions, particularly Zn^2+ ^and Cu^2+^, are involved in amyloidogenesis and other related pathways in neurodegenerative diseases [55,56].

In comparison to amyloid fibrils, the BRD filaments are of similar size to amyloid protofilaments, presumably because they share repeating parallel β-strand motifs, but they do not stain with congo red of thioflavin T and they do not associate into fibrils as readily. The BRD filaments are also easily solubilised by the addition of EDTA.

Since beginning this design project it is of interest to note that our lab has discovered a hyperactive antifreeze protein from Antarctic lake bacterium, *Marinomonas primoryensis*, that appears to be similar to RTX toxin proteins and alkaline protease from *Pseudomonas aeruginosa*. The protein is very large and contains several domains (unpublished). The domain responsible for the very high antifreeze protein activity contains a putative β-roll with GGXGXDXUX repeats, that is very similar to the alkaline protease β-roll template used to design the filament forming BRD polypeptides. Antifreeze activity is only observed in the presence of Ca^2+ ^suggesting that the domain forms a calcium-bound β-roll motif. The BRD polypeptides were originally designed with the future goal of engineering an antifreeze protein. At the time, no such β-roll AFP had been discovered but our recent findings support the use of a metal-binding β-roll peptide as a scaffold to design an antifreeze protein.

## Conclusion

In summary, polypeptides based on the calcium-binding β-roll domain of alkaline protease from *Pseudomonas aeruginosa *were designed and synthesized. In the presence of lanthanum ions, the polypeptides polymerized and formed non-amyloid-like aggregates. Analysis of this phenomenon by right angle light scattering and electron microscopy confirmed the presence of protein filaments several hundreds of nanometers in length but only 3 nm in diameter. These filaments were only formed in the presence of La^3+^, no other metal ions tested could induce filament formation. A novel feature of these filaments is their reversible nature caused by the addition of EDTA. In addition, modification of the N terminus of BRD2 with biotin produced a capping effect whereby the length and abundance of the filaments could be controlled by varying the ratio of modified to unmodified peptide.

## Methods

### Peptide synthesis and purification

Peptides were prepared by solid-phase synthesis on a PerSeptive Biosystems 9050 Plus peptide synthesizer, as peptide-amides using PAL-PEG-PS resin (PerSeptive Biosystems). A coupling procedure, employing O-(7-azabenzotriazol-1-yl)-1,1,3,3-tetramethyluronium hexafluorophosphate active esters of 9-fluorenylmethoxy-carbonyl amino acids was used. The peptides were cleaved from the resin with an 81:13:1:5 (v/v) mixture of trifluoroacetic acid, thioanisole, m-cresol, and ethanedithiol, respectively, for 30 min at 25°C. The peptides were purified by C18 reversed-phase chromatography, and peptide identity was confirmed by matrix-assisted laser desorption/ionization mass spectrometry (MALDI-MS) and amino acid analysis. Peptide purity was assessed by analytical C18 reversed-phase chromatography using the Pharmacia FPLC system. BioBRD2 was synthesized using FMOC solid phase chemistry by Dr. Nam-Chaing Wang at the Advanced Protein Technology Centre, The Hospital for Sick Children, Toronto, ON, Canada. The peptide was purified by HPLC as stated above.

### Circular dichroism spectroscopy

Circular Dichroism measurements were performed on an Aviv 62A DS Circular Dichroism Spectrometer. Spectra were obtained using a 1 mm quartz cuvette at 25°C in water or 10 mM HEPES pH 7.6. The peptide concentration for each spectra was 10 μM and the wavelength range was 190–280 nm. All CD measurements are reported in millidegrees.

### 1D NMR spectroscopy

1D NMR spectra were recorded on a Varian Inova 600 MHz NMR spectrometer equipped with a z-gradient HCN probe. Acquisition parameters were: acquisition time 2 s; sweep width 8000 Hz; recycle delay 2.5 s; number of scans 512; and a 90° pulse. Spectra are presented with a 0.5 Hz line broadening for increased signal-to-noise. The BRD1 sample was added to 90% H_2_O/10% D_2_O to make an approximately 0.5 mM solution, and the pH adjusted to 6.5. Calcium was titrated into the sample up to 128 mM.

### Right angle light scattering timedrives

Right angle light scattering was measured using a Perkin Elmer LS-50 luminescence spectrometer and FL WinLab v3.0 software. Samples were excited at 320 nm and the right angle light scattering at 320 nm at 90° to the excitation beam was collected. Slit widths were set to 2.5 nm and all experiments were carried out at room temperature (~22°C). Samples were placed in a stirred 3 mL quartz fluorescent cuvette containing 20 mM 4-(2-hydroxyethyl)-1-piperazineethanesulfonic acid (HEPES), pH 7.6 as a buffer. At 200 s a solution of BRD polypeptide(s) was injected into the cuvette usually giving a final concentration of 10 mM BRD polypeptide but other concentrations were also used. At 500 s, a 1 M stock of lanthanum trichloride was injected using a syringe pump set to 4 μL/min for a total of 120 s. At 1200 s, a 0.5 M stock solution of EDTA was injected, also at 4 μL/min until the right angle light scattering signal returned to the baseline.

### Electron microscopy

Initial samples of the BRD1 and 2 aggregates were dried for a period of a few days on charged Pioloform, carbon-coated EM grids and then negative stained with 1 % phosphotungstic acid. For subsequent EM images, negatively stained fibrils were prepared by floating peptide solutions (100 μM total peptide concentration, 10 mM La^3+ ^in 20 mM HEPES, pH 7.6) on Formvar-coated Ni^2+ ^EM grids (Pelco No. 160, Redding, CA, USA). These solutions were incubated for 3–5 days. After the grids were blotted and air-dried, the samples were stained with 1 % (w/v) phosphotungstic acid. Electron microscopy images of the peptides were acquired on a Hitachi H-7000 transmission electron microscope (TEM) operated with an accelerating voltage of 75 kV at 30,000 × magnification.

### Amino acid analysis and elemental analysis

Amino acid analysis was performed at the Advanced Protein Technology Centre, The Hospital for Sick Children, Toronto, ON, Canada. Trace element analysis for La3+ was carried out on samples using inductively coupled plasma atomic emission Spectra (ICP-AEOS) at ANALEST, Department of Chemistry, University of Toronto, ON, Canada.

### BRD polypeptide modeling

The BRD1 and 2 models were constructed and minimized using SYBYL 6.0. The structures of BRD1 and 2 were threaded onto the alkaline protease β-roll domain using Swiss-PdbViewer and SWISS-MODEL. The models of the docked BRD2 filaments were generated using HEX. Ray traced figures in this paper were generated using PyMol v0.99.

### Dynamic light scattering

DLS data were collected using a Wyatt DynaPro Titan molecular sizing system at 25°C using Dynamics software v5.26.38. The system was calibrated using ultrapure water and 2 mg/mL bovine serum albumin (BSA). Samples were prepared as above (100 μM final protein concentration) and analyzed in a 12 μL quartz cuvette. A total of 25 data points were collected for calculation of the average hydrodynamic radius for each sample.

### Thioflavin T fluorescence scans

Samples of BRD2 filaments (100 μM final peptide concentration, 10 mM La^3+^) and Aβ-40 amyloid fibrils (with and without 10 mM La^3+^) were assayed for thioflavin T (ThT) binding (0.035 mg/mL) using a Perkin Elmer LS-50 luminescence spectrometer and FL WinLab v3.0 software. Samples were excited at 455 nm in stirred 1 mL quartz fluorescent cuvettes and spectra were collected between 460 nm and 560 nm (Slit widths = 2.5 nm).

### Congo Red UV/Visible spectrometry

Samples of BRD2 (100 μM final peptide concentration) with and without La^3+ ^(10 mM final concentration) and serum amyloid A fibrils purified from rat pancreas were allowed to bind to freshly prepared Congo red (7 μM) overnight in a 1 mL disposable cuvette. The samples were mixed and the spectrum from 400 nm to 600 nm was recorded on a Cary 50 Bio UV spectrophotometer running Cary WinUV v2.0 software.

## Abbreviations

BRD1 and 2: β-roll designed polypeptides 1 and 2; CAST: full length calpastatin; CD: circular dichroism; DLS: dynamic right angle light scattering; EDTA: ethylenediaminetetraacetic acid; EM: electron microscopy; HEPES: 4-(2-hydroxyethyl)-1-piperazineethanesulfonic acid; NMR: nuclear magnetic resonance; PGAL: 6-phospho-β-galactosidase; RTX: repeat-in-toxin

## Competing interests

The author(s) declares that there are no competing interests.

## Authors' contributions

RLC, MMT and PLD designed the BRD polypeptides. Peptides were synthesized and purified by MG and AC. CD data were collected by MG, MMT and AC. 1D NMR data were collected by MED and BDS. Right angle light scattering and dynamic light scattering data were collected by AJS. EM images were collected by AJS and RJO. Congo red and thioflavin T data were collected by AJS, DAB and AC. The model of the BRD2 filaments was prepared by RLC. All the authors read and approved the manuscript.
